# Possible thoracic metastasis from squamous cell carcinoma of the external auditory canal: A case report

**DOI:** 10.1002/ccr3.9459

**Published:** 2024-09-16

**Authors:** Hiroshi Takehara, Ken Kodama, Toru Momozane, Masashi Takeda, Kaichi Shigetsu, Hiroki Kishima

**Affiliations:** ^1^ Department of Thoracic Surgery Yao Municipal Hospital Yao Japan; ^2^ Department of Pathology Yao Municipal Hospital Yao Japan; ^3^ Department of General Thoracic Surgery Osaka University Osaka Japan; ^4^ Department of Surgery Kishima Hospital, Main Division Yao Japan

**Keywords:** external auditory canal, pembrolizumab, squamous cell carcinoma, thoracic metastasis

## Abstract

An 80‐year‐old never‐smoking woman, who underwent radiotherapy in combination with simultaneous intra‐arterial chemotherapy for external auditory canal squamous cell carcinoma (SCC) 10 years ago, was referred to our department due to a painful huge chest wall tumor. We conducted surgical resection combined with the right upper lobe and chest wall including the 3rd to 5th ribs and affected serratus anterior muscle. Histologically, atypical keratotic cells with the same morphology as external auditory canal SCC proliferated without the figure of carcinoma in situ or squamous dysplasia of the bronchial epithelium of the lung parenchyma adjacent to the tumor. **Take Home Message:** If long‐term imaging follow‐up had been performed, it would have been easier to detect it early and differentiate it from metastasis or primary lung cancer.

## 1 INTRODUCTION

Cancer of the external auditory canal (EAC) is rare, representing less than 0.2% of all head and neck cancer.^1^ Squamous cell carcinoma (SCC) is the most common neoplasm at this site, followed by basal cell carcinoma (BCC) and adenoid cystic carcinoma (ACC). The structure frequently involves cutaneous SCC of the pinna.^2^ The prognosis of the patients classified as T4 (Pittsburg classification) is poor and most patients die of the disease, especially due to loco‐regional recurrences within 2 years, regardless of treatment modalities. Distant metastases during the course of the disease are observed in about 10%.^3^

We encountered a patient who developed a painful tumor in the chest wall and underwent an operation 10 years after treatment for EAC SCC. The resected tumor was probably a late thoracic metastasis of EAC SCC. The clinical course and pathological findings are discussed.

## 2 CASE HISTORY

**FIGURE 1:**
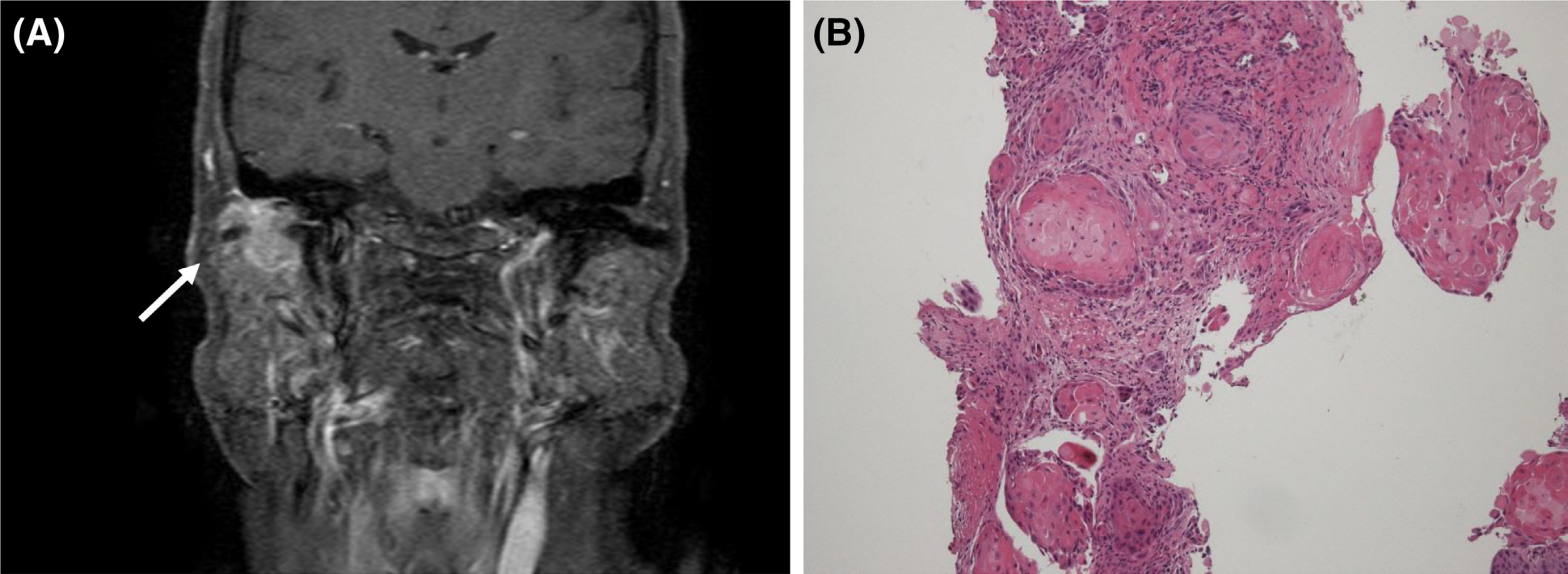
(A) Enhanced MRI showing a 20‐mm mass lesion (arrow) extending from the right external auditory canal to upper part of the parotid gland. Anteriorly, it contacts the articular process of the mandible. The contrast dye enhances the entire tumor. The tumor is suspected to have invaded the parotid gland. (B) The biopsy specimen. A histological examination showing well‐differentiated invasive squamous cell carcinoma with intracytoplasmic keratin (hematoxylin–eosin staining, object lens 10×).

An 80‐year‐old Japanese woman with a prior history of treatment for right EAC SCC (Figure 1) 10 years ago was referred to our department due to a painful right chest tumor.

At the age of 69 years old, she had received radiotherapy in combination with simultaneous intra‐arterial chemotherapy (RADPLAT) followed by 2 cycles of intravenous cisplatin and docetaxel^4^ for the EAC SCC (Pittsburgh Classification: T4N0M0) at the Department of Otorhinolaryngology of the university hospital and our hospital, and a complete response (CR) was achieved. The patient was reported to be a never‐smoker with no history of asbestos exposure. Also, she was not exposed to second‐hand smoke daily in her home. In addition, the patient had no history of cryosurgical treatment for skin lesions.

## 3 EXAMINATION

**FIGURE 2:**
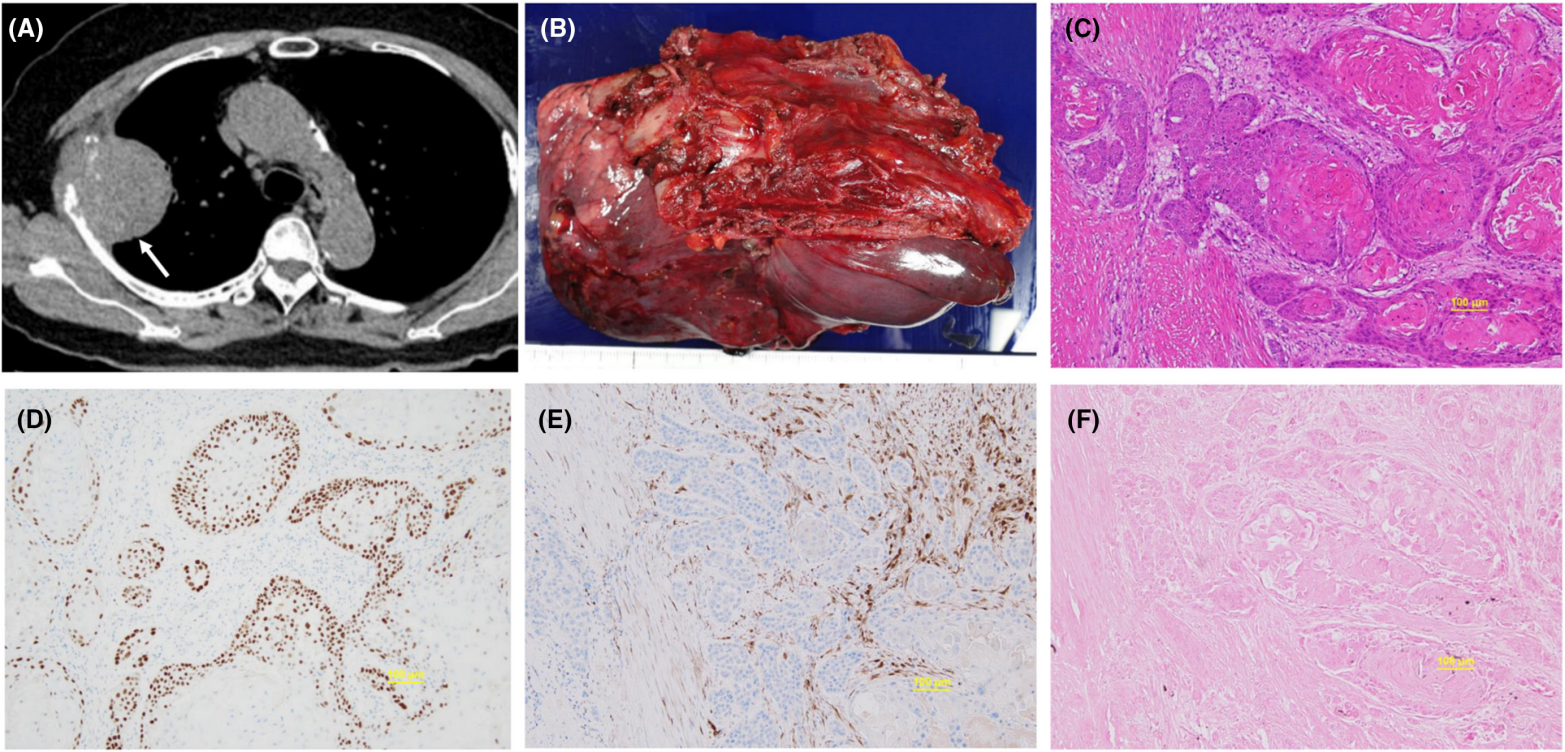
(A) Chest CT showing a 60‐mm well‐demarcated mass lesion (arrow) with bidirectional extension to the chest wall and lung, and no cavity formation. (B) Resected specimen including the upper lobe of the right lung and 3rd‐5th ribs. Macroscopic margin cancer‐free. (C) Resected specimen. Histological examination showing well‐differentiated squamous cell carcinoma with intracytoplasmic keratin and pearls (hematoxylin–eosin staining, object lens 10×). (D) Diffusely positive immunohistochemical staining (IHS) for *P53* (object lens 10×). (E) Negative IHS for p16. (F) In situ hybridization (ISH) for Epstein–Barr virus is negative (object lens 10×).

A roentgenogram and computed tomography (CT) revealed a mass larger than 6 cm showing bidirectional extension toward the chest wall and right upper lobe of the lung (Figure 2A). Positron emission tomography (PET) revealed no abnormal uptake of fluorine‐18 2‐deoxy‐2‐fluoro‐D‐glucose (FDG) except in the chest tumor. Brain magnetic resonance imaging (MRI) revealed neither local recurrence of EAC nor brain metastasis. The SCC was proven by echo‐guided percutaneous aspiration cytology of the chest tumor. Serum levels of cytokeratin‐19 fragment and SCC antigen were elevated to 3.7 ng/mL (normal, <3.5 ng/mL) and 8.6 ng/mL (normal, <2.5 ng/mL), respectively. The remainder of her laboratory data were within normal limits.

## 4 METHODS

Based on the clinical diagnosis of T3N0M0 lung cancer, we conducted surgical resection combined with the right upper lobe and chest wall including the 3rd–5th ribs and affected serratus anterior muscle with a safe margin of about 2 cm (Figure 2B). Intrathoracic procedures were performed through a lateral defect of the chest wall resulting from the resection of the chest wall. Systematic hilar and mediastinal lymph node dissection was performed. In addition, a small nodule discovered on the visceral pleura of the middle lobe was removed. The chest wall defect was reconstructed with 10 × 8‐cm large, 2‐mm thick ePTFE GORE® DUALMESH®.

## 5 OUTCOME

Macroscopically, the main tumor that was excised was 8.5 × 6.0 × 5.0 cm, with clear borders, grayish white, and a solid appearance (Figure 2B). Histologically, atypical keratotic cells with swollen nuclei and conspicuous nucleoli, ranging from round to mildly irregular, proliferated and exhibited a solid or small‐sized cluster growth pattern (Figure 2C). The tumor invaded the pleura, 3 ribs, and serratus anterior muscle. It could be seen infiltrating the interlobar pleura and exposed on the pleural surface of the upper lobe. Although no obvious lymphatic invasion was noted in the specimen, numerous vascular invasions were observed. However, neither SCC in situ nor dysplasia was observed in the bronchial epithelium of the lung parenchyma adjacent to the tumor. Based on the gross and histological findings, the epicenter of the tumor was considered to be in the chest wall. The resected nodule on the middle lobe pleura was proven to be the same keratinizing SCC. There was no metastatic lesion of the dissected lymph nodes. Epidermal growth factor receptor (EGFR) was wild‐type, and the tumor cells expressed programmed death‐ligand 1 (PD‐L1) at a rate of <1%. P53 and p16, a surrogate marker of human papilloma virus infection, were negative on immune histochemical staining (IHS) (Figure 2D,E). The in situ hybridization (ISH) test for Epstein–Barr virus (EBV) encoded small nuclear RNAs (EBERs) was also negative (Figure 2F).

## 6 FOLLOW‐UP

**FIGURE 3:**
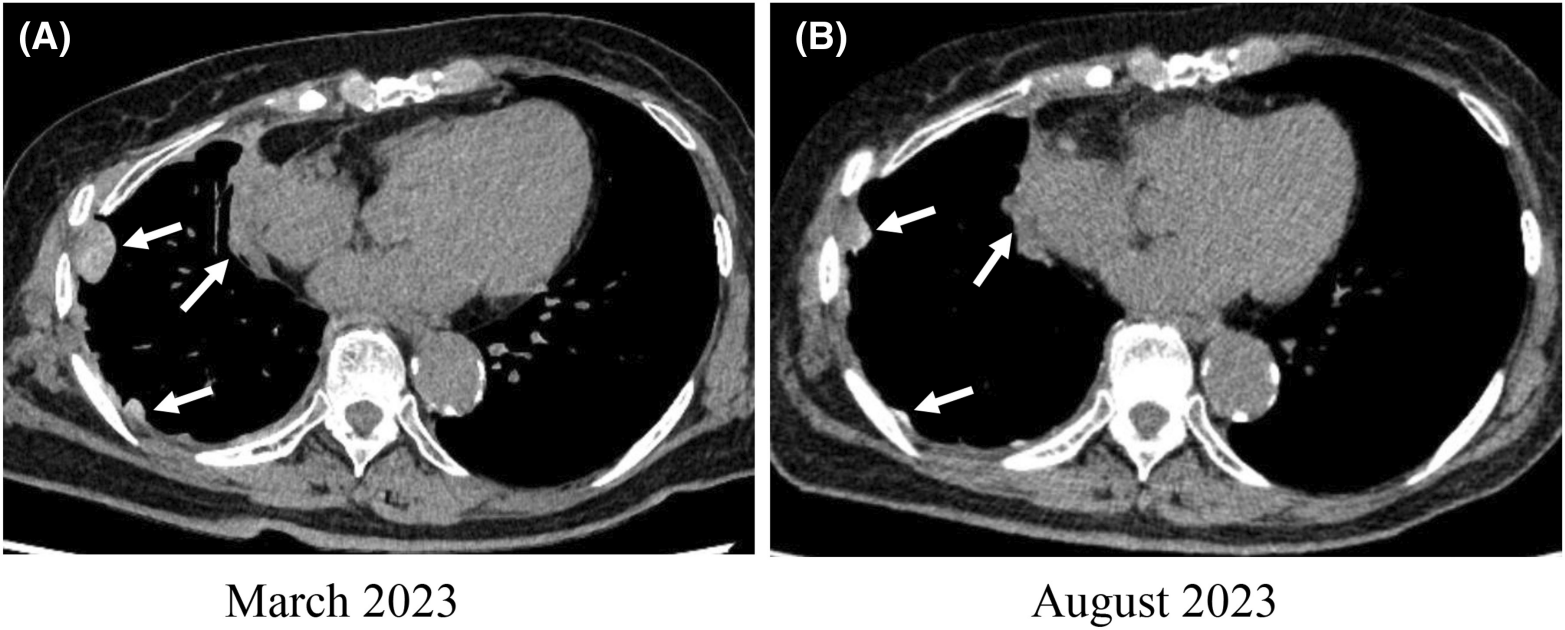
Chest CT taken before (A) and after (B) five courses of pembrolizumab administration. A partial response for pleural recurrence (arrows) was achieved.

The post‐operative course was uneventful. However, pleural metastasis was observed by CT taken 5 months after surgery (Figure 3A). The Eastern Cooperative Oncology Group (ECOG) performance status (PS) score was 1. We started pembrolizumab combined with carboplatin and nab‐paclitaxel. Considering the advanced age of the patient, monotherapy of pembrolizumab every 3 weeks was employed from the 2nd course, achieving disease control with a partial response (Figure 3B). However, after 11 courses of pembrolizumab, immune‐related cardiac toxicity with massive pericardial effusion, and dyspnea developed. Thus, pembrolizumab was discontinued, and prednisolone at 45 mg/day was administered in combination with furosemide. The patient remains alive and asymptomatic with stable disease 15 months after the surgery. Loco‐regional recurrence at the EAC site has not been detected to date.

## 7 DISCUSSION

Temporal bone SCC includes both EAC and middle‐ear SCC, and it is unclear whether it should be treated as a type of skin cancer or in the same way as other SCC of the head and neck in clinical practice. When focusing on EAC, SCC generally shows more aggressive behavior and is associated with a poorer prognosis than other tumors of the auditory canal. The overall 5‐year survival rate has been reported to be 37%, and survival for stage IV (T4 and any TN+) patients was 16%.^5^ Prognostic factors for carcinoma of EAC are: TNM stage, bony erosion of EAC, positive surgical margins, extratemporal locoregional invasion (parotid, cervical), middle‐ear involvement, and presence of peripheral facial nerve palsy.^3^

According to a recent meta‐analysis, the local recurrence rate of EAC SCC was 32.3%, nodal recurrence was 3.9%, and distant metastasis was 0.7%. Locally advanced tumors were associated with a poor prognosis. Poor outcomes mostly occurred due to local recurrence.^6^ Because of the extremely low prevalence, there have been no data concerning the treatment and survival of patients with distant metastasis. According to our literature survey, there has been only one case report of EAC SCC with surgical treatment of pulmonary metastasis.^7^

In the present case, the resected specimen combined with the chest wall and lung exhibited the same pathologic appearance as that of EAC treated 10 years ago. It showed well‐differentiated SCC with a large amount of keratin. Unfortunately, no histological examination other than hematoxylin–eosin (H‐E) staining was performed of biopsy tissue from EAC SCC, but the chest tumor that was excised this time was negative for EBER‐ISH and p16 IHS, and diffusely positive for p53 IHS. Therefore, it exhibited characteristics more similar to cutaneous SCC than head and neck SCC. Next, there is the issue of differentiating between SCC that has developed in the peripheral lungs and late metastasis of EAC SCC. This female patient is a never‐smoker who lives without a risk of exposure to second‐hand smoke. As a result, there has been neither cystic change, interstitial fibrosis, nor anthracosis in the background lung, which are significantly correlated with peripheral‐type primary lung SSC.^8^ Furthermore, based on the imaging and intraoperative findings, the tumor appears to have arisen from the chest wall rather than from the lungs. Pathologically, there was no evidence of SCC or squamous metaplasia extending to the bronchial epithelium around the tumor. The lung SCC of never‐smokers were more often reported as poorly differentiated.^9^ Further, 40%–60% of non‐small cell lung cancer (NSCLC) tumors are reportedly associated with mutations of the tumor suppressor gene p53, and these mutations are more common in tobacco‐associated lung cancer than in lung cancer in never‐smokers.^10^ Our case is a never‐smoker, and the positive p53 IHS suggests the possibility of metastasis from EAC SCC, which has the characteristics of cutaneous SCC. Based on these facts, the lesion resected from the chest wall was considered to be very late hematogenous metastasis to the chest wall after treatment for EAC SCC.

We encountered unexpected pleural dissemination with single visible node on the middle lobe surface without malignant effusion intraoperatively. In addition, we confirmed that pleural lavage cytology at thoracotomy was negative. Recent study indicated that main tumor and visual nodule resection could improve overall survival and progression‐free survival for the NSCLC patients with the unexpected pleural dissemination.^11^ During surgery, we performed surgery based on the diagnosis of T3N0M0 NSCLC. We determined that upper lobectomy combined chest wall resection including the nodule in the middle lobe was appropriate for survival and pain control. Hu et al.^11^ concluded that sublobar resection without systematic lymphadenectomy may be the optimal procedure in such case. Our case was concluded at final diagnosis to be possible metastasis of EAC SCC, and the tumor was large, making sublobar resection difficult. In addition, because this is an extremely rare case, it remains unclear whether systematic lymphadenectomy should be performed in such case.

There has been no evidence‐based follow‐up schedules or treatment modalities for metastasis after EAC SCC treatment, because of the fact that the vast majority of EAC SCC recurrences are local. Immunotherapy is emerging as an important arm of cancer therapy, especially for advanced cutaneous SCC of the head and neck, yet only case reports have been published on its use for ear canal cancer.^12^ Based on the report of Sano, et al.,^13^ we used pembrolizumab for the treatment of pleural dissemination after removal of the chest lesion, and control has been possible for more than 10 months.

## 8 CONCLUSION

We resected a right chest tumor in a patient who had undergone RADPLAT for T4 right EAC SCC 10 years earlier. Based on the clinical and pathological findings, very late metastasis of EAC SCC to the chest wall was strongly suspected, but a definitive diagnosis has yet to be made. Our experience suggests that even if advanced EAC SCC is considered as showing a CR to treatment, periodic long‐term follow‐up including chest imaging is necessary.

## AUTHOR CONTRIBUTIONS

**Hiroshi Takehara:** Conceptualization; investigation; resources; validation; writing – original draft; writing – review and editing. **Ken Kodama:** Conceptualization; investigation; resources; supervision; writing – original draft; writing – review and editing. **Toru Momozane:** Investigation; resources; writing – review and editing. **Masashi Takeda:** Investigation; resources; writing – review and editing. **Kaichi Shigetsu:** Investigation; resources; writing – review and editing. **Hiroki Kishima:** Investigation; resources; writing – review and editing.

## FUNDING INFORMATION

None of the authors has any financial disclosures.

## CONFLICT OF INTEREST STATEMENT

All authors declare no conflict of interest.

## ETHICS STATEMENT

This report was approved by the ethics committee of the Yao Municipal Hospital (Approval no. YMH220324‐203).

## CONSENT

Written informed consent was obtained from the patient to publish this report in accordance with the journal's patient consent policy.

## Data Availability

The data that support the findings of this study are available on request from the corresponding author. The data are not publicly available due to privacy or ethical restrictions.
